# A Calcium Guard in the Outer Membrane: Is VDAC a Regulated Gatekeeper of Mitochondrial Calcium Uptake?

**DOI:** 10.3390/ijms22020946

**Published:** 2021-01-19

**Authors:** Paulina Sander, Thomas Gudermann, Johann Schredelseker

**Affiliations:** 1 Walther Straub Institute of Pharmacology and Toxicology, Faculty of Medicine, LMU Munich, 80336 Munich, Germany; paulina.sander@campus.lmu.de (P.S.); thomas.gudermann@lrz.uni-muenchen.de (T.G.); 2 Deutsches Zentrum für Herz-Kreislauf-Forschung, Partner Site Munich Heart Alliance, Munich, Germany

**Keywords:** mitochondrial calcium uptake, VDAC, MCU

## Abstract

Already in the early 1960s, researchers noted the potential of mitochondria to take up large amounts of Ca^2+^. However, the physiological role and the molecular identity of the mitochondrial Ca^2+^ uptake mechanisms remained elusive for a long time. The identification of the individual components of the mitochondrial calcium uniporter complex (MCUC) in the inner mitochondrial membrane in 2011 started a new era of research on mitochondrial Ca^2+^ uptake. Today, many studies investigate mitochondrial Ca^2+^ uptake with a strong focus on function, regulation, and localization of the MCUC. However, on its way into mitochondria Ca^2+^ has to pass two membranes, and the first barrier before even reaching the MCUC is the outer mitochondrial membrane (OMM). The common opinion is that the OMM is freely permeable to Ca^2+^. This idea is supported by the presence of a high density of voltage-dependent anion channels (VDACs) in the OMM, forming large Ca^2+^ permeable pores. However, several reports challenge this idea and describe VDAC as a regulated Ca^2+^ channel. In line with this idea is the notion that its Ca^2+^ selectivity depends on the open state of the channel, and its gating behavior can be modified by interaction with partner proteins, metabolites, or small synthetic molecules. Furthermore, mitochondrial Ca^2+^ uptake is controlled by the localization of VDAC through scaffolding proteins, which anchor VDAC to ER/SR calcium release channels. This review will discuss the possibility that VDAC serves as a physiological regulator of mitochondrial Ca^2+^ uptake in the OMM.

## 1. Introduction

Mitochondria are known as the cell’s powerhouses as they produce ATP by oxidative phosphorylation. However, already in the 1950s and 1960s, researchers noted the potential of mitochondria to sequester vast amounts of Ca^2+^ [1,2], while the role of this mitochondrial Ca^2+^ uptake and the involved molecular players remained elusive. Many proteins including RyR, LetM1, or UCP2/3 had been suggested to mediate mitochondrial Ca^2+^ uptake, before the molecular identity of the MCU complex (MCUC) in the inner mitochondrial membrane was identified as the main route of Ca^2+^ uptake in the early 2010s. Subsequently, research on mitochondrial Ca^2+^ uptake experienced a revival when molecular structures and knock-out models of the individual MCUC components became available. However, on its way into mitochondria Ca^2+^ has to pass two membranes, and the first barrier before Ca^2+^ even reaches the MCUC is the outer mitochondrial membrane (OMM), a fact that is still often neglected in current literature, where the OMM is described as freely permeable to ions. Indeed, the term mitochondrial Ca^2+^ uniport is often used synonymously for mitochondrial Ca^2+^ uptake, and some textbook representations do not even show the OMM but depict the MCUC such that it is directly facing the cytosol.

The OMM is widely occupied by large pore-forming structures, the voltage-dependent anion channels (VDACs), which mediate the flux of ions and metabolites over the OMM. While bacteria express only one form of VDAC, higher eukaryotes express multiple isoforms. In yeast, two isoforms are described, while plants and animals express three isoforms (VDAC1-3).

In a ground-setting publication in 2001, Shoshan-Barmatz and colleagues provided several lines of evidence that the voltage-dependent anion channel (VDAC) is the main carrier for Ca^2+^ over the OMM [3]: when inserted into planar lipid bilayers, VDAC1 was permeable to Ca^2+^, and VDAC1 reconstituted into liposomes mediated Ca^2+^ efflux from these vesicles. These in situ results are supported by the observations that VDAC1 overexpression enhanced Ca^2+^ uptake into mitochondria [4], and knock-down of VDAC1 reduced mitochondrial Ca^2+^ uptake [5] in vitro in cultured cells. Already in these early publications, it was noted that the Ca^2+^ flux through VDAC could be blocked by ruthenium red (RuR) and La^3+^ and that VDAC possesses a Ca^2+^ binding site, raising the intriguing hypothesis that VDAC is not freely permeable to Ca^2+^ but might present a regulated barrier for Ca^2+^ flux over the OMM.

However, considering that VDAC forms a pore large enough to allow the passage of metabolites such as ATP, two questions arise: how can such a big pore be regulated at the molecular level and is the regulation of mitochondrial Ca^2+^ uptake at the outer mitochondrial membrane physiologically relevant?

Several recent reports have argued in favor of a regulation of mitochondrial Ca^2+^ uptake at the OMM: (i) the tight connection between VDAC1 and the IP3 receptor (IP3R) was reported to be a key regulatory mechanism to either promote enhanced cellular metabolism or to induce apoptosis by modulating mitochondrial Ca^2+^ signaling [5,6], (ii) regulation of VDAC-mediated Ca^2+^ uptake through channel monoubiquitinylation [7] was demonstrated to be critically involved in the pathophysiology of Parkinson’s disease and to mediate programmed cell death after DNA damage [8], and iii) our lab has recently demonstrated that the small compound efsevin modulates VDAC2 and, thereby, amplifies mitochondrial Ca^2+^ uptake approx. 3-fold in cardiomyocytes [9,10]. This amplification induces a protective effect against cardiac arrhythmia, both in vitro and in vivo.

In this review, we will first discuss possible regulatory mechanisms of mitochondrial Ca^2+^ uptake through VDAC and then review their putative roles in cell physiology and pathophysiology.

## 2. Mechanisms to Control the Ca^2+^ Conductance of VDAC

### 2.1. Structure and Electrophysiological Properties of VDACs

**Figure 1:**
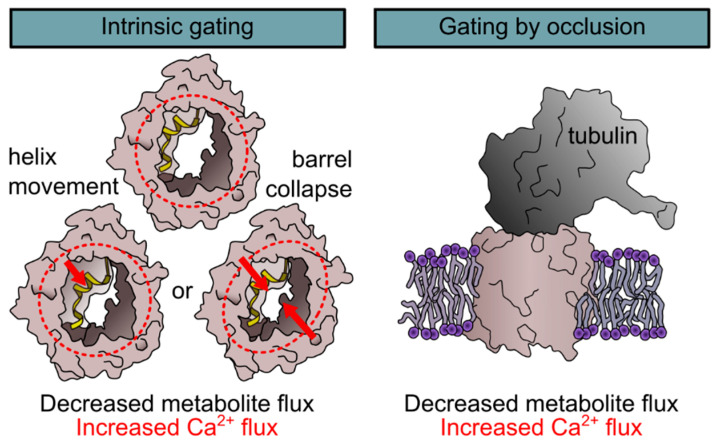
Mechanisms of voltage-dependent anion channels (VDAC) gating. Two mechanisms for the gating of VDACs were described, namely gating by an intrinsic conformational change (left) and gating by occlusion (right). The first is experimentally induced by voltage but is modulated by extrinsic parameters such as protein interactions, the lipidic environment or posttranslational modifications. Intrinsic gating results in a conformational change of a yet unknown nature. Several models ranging from a movement of the N-terminal α-helix to a collapse of the barrel were suggested. The second mechanism that was observed for VDAC gating is gating by occlusion by, e.g., free tubulin through a molecular plug model. Both mechanisms were shown to block metabolite flux and to induce a lower anion selectivity and, thus, an increased Ca^2+^ flux.

**Figure 2:**
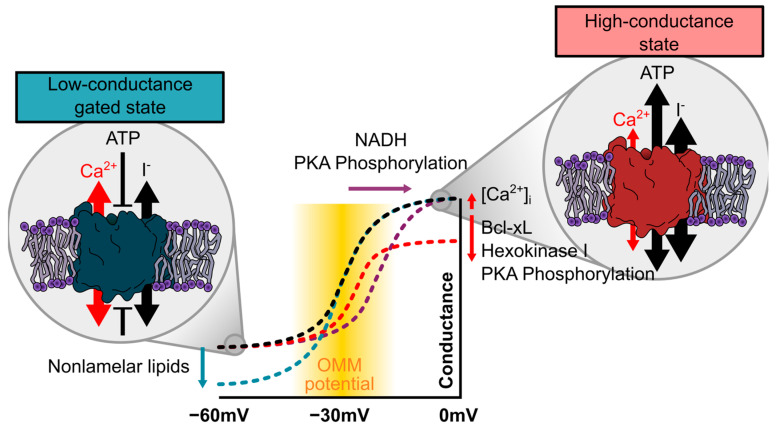
Regulation of voltage-dependent gating of VDAC. While VDAC almost exclusively resides in its anion-selective and metabolite-permeable high-conductance state at neutral potentials, it starts gating within the range of −20 to −30 mV (black line), which is well within the predicted range of an OMM potential (yellow zone). Note that the conductance–voltage relation of VDAC is symmetrical, and only the negative side is depicted. At potentials below −40 mV, it almost exclusively resides in the low-conductance state that is impermeable for ATP but shows a higher permeability of Ca^2+^. Several factors were reported to modulate this voltage–conductance relationship. The phosphorylation status or binding of protein partners such as Bcl-xL or hexokinase were suggested to facilitate channel gating and, thus, induce both a right-shift of the voltage dependence and a reduction in the maximum conductance at potentials close to neutral (red line), while Ca^2+^ is suggested to facilitate channel opening and, thus, has an opposite effect. Nonlamellar lipids were demonstrated to promote channel closure at very negative potentials (blue).

The common perception that VDAC creates pores in the OMM that are freely permeable to ions is closely related to its structure. VDACs are 30–35 kD transmembrane proteins consisting of approximately 280–300 amino acids, depending on isoform and species. They form barrel-like pores consisting of 19 β-sheets aligned in an antiparallel orientation. On the N-terminal end, an α-helix lines the channel pore inside the barrel [11,12]. The odd-numbered 19 β-sheet geometry is unique for pores within the OMM and was postulated to possess superior properties regarding voltage sensitivity, mitochondrial targeting, and lipid-modulated stability over barrels with a higher or lower number of sheets [13]. The pore has a diameter of approx. 18–20Å [14]. Nevertheless, despite this large diameter, the channel can undergo conformational changes of a still unknown structural nature, which induce a significant change in the channel’s conductance and ion selectivity. When purified VDAC is inserted into artificial lipid bilayers the channel is in a high-conductance state at neutral potential that is classically also referred to as the open state. Upon gradual polarization of the membrane, the channel starts gating at around ±20–30mV between this high-conductance state and several gated states, which are also referred to as closed states (Figure 1 and Figure 2). In these states, the conductance of the channel is reduced to approximately 50% [15,16,17,18]. By using CaCl_2_ as the charge carrier, the channel was shown to be permeable for Ca^2+^. Interestingly, the ion selectivity of the channel changes upon gating from a higher anion selectivity in the high-conductance state to a lower selectivity and, thus, higher proportional conductance for Ca^2+^ in the gated states [3,19,20,21]. Though this observation is consistent, the degree of ion selectivity varies between distinct reports and experimental conditions depending on, e.g., the concentration of CaCl_2_ used or the composition of the bilayer making an estimation of VDACs ion selectivity in the native environment in the cell difficult. Nevertheless, the relative permeability of cations over anions in VDAC1 was identified experimentally and by molecular simulations to be in the range of 0.05 to 0.4 for the high-conductance state, while the relative permeability for cations over anions was almost 1 for the gated states [3,19,20,21] for VDAC1 and VDAC2 [10]. Ambiguity exists concerning the electrophysiological properties of VDAC3. While initially reported to form channels with low conductance and weak voltage-dependent gating [22], this isoform was later reported to have similar electrophysiological properties as VDAC1 and VDAC2 [23]. However, to our knowledge, no reports are available for the ion selectivity and Ca^2+^ conductance of VDAC3.

The transition between high-conductance and gated states induces drastic changes in the permeability and selectivity. Although, lipid bilayer experiments are performed in an artificial and rather unphysiological system and care must be taken when transferring them directly to the in vivo situation, it is conceivable that gating of the channel presents a mechanism to regulate not only ATP, but also Ca^2+^ flux over the OMM. However, gating of the channel was experimentally induced by voltage, and although several mechanisms to create and regulate a membrane potential over the OMM were postulated [24,25,26], it remains controversial whether this is a physiologically relevant trigger of gating. Several other factors including Ca^2+^ [27], metabolites [28], the lipidic environment [29,30,31,32], temperature [33], small molecules [10,34,35], interacting proteins [6,36,37], and biochemical modifications of the channel itself [38,39] were reported to either modify voltage-induced gating or to induce channel closure on their own. Among those is also the intriguing hypothesis of an entirely different mode of channel gating, namely a closure by occlusion of the large VDAC pore by a “molecular plug” mechanism (Figure 1). In the following, we will discuss the different modes of gating in respect to the Ca^2+^ conductance of the channel.

#### 2.1.1. Regulation of Ca^2+^ Conductance by Gating

Evidence for the ability of the channel to undergo intrinsic conformational changes for gating was provided by lipid bilayer experiments on purified channels [15,16]. VDAC heterologously expressed in *E. coli* and, subsequently, purified and refolded from inclusion bodies forms functional channels in lipid bilayers, which are completely devoid of any regulatory partners that might be associated with the channel in its native environment [10,11]. These channels are able to reversibly gate between the high-conductance and gated states indicating that gating is an intrinsic property of the channel protein. Various models were proposed to explain the molecular movements underlying gating of VDACs (Figure 1). Many of these models involve participation of the N-terminal α-helix and include mechanisms with relatively small movements of the helix inside the barrel [17,40] and models in which the helix moves outside of the barrel and induces its collapse leading to channel closure [20,41]. However, while deletion of the helix abrogated voltage-gating of the channel, cross-linking of the helix against the channel wall did not [42]. This favors a model in which the helix stabilizes the barrel, while gating is induced by a yet uncharacterized deformation of the barrel.

As described earlier, gating of the channel was demonstrated to directly affect its Ca^2+^ conductance, hence gating would be an intriguing possibility to regulate VDAC Ca^2+^ flow. However, the existence of voltage-dependent gating in the native environment remains controversial. Several extrinsic factors were described to influence and modulate voltage-gating of the channel and are discussed later.

#### 2.1.2. Regulation of Ca^2+^ Conductance by Occlusion

In addition to the intrinsic gating of the channel, another intriguing hypothesis that was more recently developed is a regulation of the channel by occlusion. Given the large pore size of the channel, which is wide enough for the passage of metabolites, it appears conceivable that metabolites or domains of interacting proteins enter the channel to serve as “channel plugs”. Among those suggested to serve as molecular plugs is NADH: while it was known for long that NADH closes VDAC [28,43], it was only recently found in a combination of high-resolution NMR with molecular dynamics simulations that NADH reduces conduction sterically by binding into the open pore at the hinge of the pore lining helix without a structural change of the channel [44]. Other partners suggested to mediate channel occlusion are free tubulin, which was demonstrated to interact with VDAC though its C-terminus [45,46,47] (Figure 1), and α-synuclein, which blocks the channel by occlusion but also translocates through the channel [48]. Although to our knowledge, no reports have selectively evaluated the influence of channel occlusion on Ca^2+^ specifically, the occluded state was also demonstrated to make the channel more cation selective [49], thus suggesting a higher Ca^2+^ permeability.

Interestingly, a recent study using NADH measurements combined with mathematical modeling has proposed that in cardiac cells 98% of all VDACs reside in a state that is impermeable for ATP, but it remains speculative whether this is the gated or occluded state [50]. It raises, however, the interesting possibility that 98% of VDACs are in a Ca^2+^ conducting state.

### 2.2. Modification of the Gating Profile of VDAC

In the following, we will summarize factors that were discussed as possible physiological triggers that control gating or occlusion of the channel.

#### 2.2.1. An OMM Potential as a Trigger for VDAC Gating

The most straight-forward idea when bearing the lipid bilayer experiments in mind is a regulation of the channel by voltage. Since VDAC resides in its high-conductance state at 0 mV and only starts gating at potentials above 20 to 30 or below −20 to −30 mV, a physiologically relevant voltage-dependent gating of the apo-form of the channel would depend on a membrane potential across the OMM. Although the generation of a membrane potential has often been considered impossible due to the high abundance and high conductance of VDACs in the OMM, a series of modeling studies by Victor and Sergy Lemeshko provides reasonable evidence for a membrane potential over the OMM. This potential was suggested to be created by a spatial separation of VDAC from the electron transport chain and to be regulated through the local concentration of ADP within the contact sites and the Gibbs free energy of the hexokinase reaction bound to VDAC [51]. Most strikingly, it was calculated to be well within the range of VDAC gating [24,52,53]. Experimental studies using pH indicators selectively targeted into the intermembrane space (IMS) have found a pH in the IMS, which is approximately 0.5 to 0.7 lower than in the cytosol, suggesting an even more negative potential of approx. −40 mV and acidification of the IMS as a modulator of the membrane potential [26].

Another hypothesis suggests that at points where the IMM is in close contact to the OMM [54], possibly at sites of VDAC and MCUC interaction [55], the strong negative potential of the IMM induces a local OMM potential by capacitative coupling between the two membranes, which allows voltage-gating of VDAC [25]. This is particularly interesting, since these contact sites are believed to be the sites of mitochondrial Ca^2+^ uptake (see Section 2.4) and closure of the channel by a local negative potential could induce a higher Ca^2+^ conductance specifically in these areas.

Taken together, such a potential difference in the OMM could very likely be at least one factor contributing to the regulation of the channel. It is feasible that voltage induces gating of the channel, while interaction partners and channel modifications shift the channels open probability towards a preferentially high-conductance or gated state (Figure 2).

#### 2.2.2. Effects of Ca^2+^ on VDAC Gating

Besides conducting Ca^2+^, several lines of evidence suggest that the channel is also regulated by Ca^2+^ and that Ca^2+^ can regulate its Ca^2+^ conductance. In their 2001 report, Shoshan-Barmatz and colleagues were among the first to suggest that VDAC1 has a Ca^2+^ binding site. This was based on several lines of evidence: (i) a Ca^2+^ induced shift in electrophoretic mobility of VDAC1, (ii) a blockade of the channel by La^3+^, which could be reversed by the addition of EGTA, and (iii) an inhibition of the channel by ruthenium derivatives such as RuR, Ru360 [3], or AzRu [56] that was reversed by the addition of Ca^2+^. More recently, a combination of NMR and single-molecule force spectroscopy revealed that VDAC barrels are highly flexible, but a significant reduction in conformational variability is induced when Ca^2+^ is bound to the channel [57]. In a very detailed work, the lab of György Hajnóczky showed that the channel resides in a non-conducting state, with openings to the low-conductance states in the absence of Ca^2+^, but starts gating and mainly resides in the high-conductance state as the concentration of Ca^2+^ increases [27], indicating a regulation of the channel through Ca^2+^. Indeed, the relationship between the external Ca^2+^ concentration and the uptake rate of Ca^2+^ into VDAC liposomes follows a non-linear relation, indicating the presence of a Ca^2+^-regulated Ca^2+^ flux through VDAC [27].

In an effort to localize the Ca^2+^ binding site, it was observed that the addition of an excess amount of Ca^2+^ prevented the blocking effect of RuR, which was reinstalled after the addition of EGTA. This suggests that ruthenium derivatives compete with Ca^2+^ for the same binding site [56]. Mutation of a glutamate residue at position 73 (E73) eliminated the blocking effect of RuR, establishing this residue as a primary candidate for the Ca^2+^-mediated control. However, E73 is located on the outside of the barrel facing the lipidic environment [11,12], and although molecular simulations predict a thinning of the nearby membrane due to the charged nature of E73 [58], it remains controversial whether Ca^2+^ can enter the lipidic environment of the membrane. Alternatively, the mere presence of E73 could stabilize the channel in a conformation that is required for Ca^2+^-mediated channel regulation.

Nevertheless, a regulation of the channels Ca^2+^ transport activity by E73 was recently confirmed in physiological experiments, where Ca^2+^ transfer from lysosomes into mitochondria after lysosomal Ca^2+^ release through TRPML1 was shown to depend on E73 [59]. In agreement with this, Ca^2+^ transfer from the SR into mitochondria in cardiomyocytes is also regulated through E73 [60]. These findings again highlight the presence of a Ca^2+^ regulated uptake of Ca^2+^ through VDAC.

#### 2.2.3. Modulation of VDAC Gating by Posttranslational Modification

A common way to regulate ion channels are posttranslational modifications. Phosphorylation of all three VDAC isoforms was repeatedly reported, and several studies showed that PKA phosphorylation results in altered gating properties of VDAC. Interestingly, PKA phosphorylation affected channel gating asymmetrically with no influence at positive potentials but a reduction in both single channel conductance and open probability at negative potentials [61,62]. This is in line with the idea that, if a potential is established at all across the outer membrane, this would be a negative potential. Similar results were obtained for phosphorylation of VDAC by c-Jun N-terminal Kinase-3 (JNK3), which also led to a lower single-channel conductance and a reduction in open probability [63]. Although the authors of these studies only delineate a role of VDAC phosphorylation for the regulation of apoptosis, the modulatory effect of VDAC on apoptosis was critically linked to its Ca^2+^ conductance [5]. In later studies, both PKA-phosphorylation mediated by the 18 kDa translocator protein TSPO [64] and phosphorylation by PKCε reduced mitochondrial Ca^2+^ accumulation [65].

These experiments are difficult to interpret. Bilayer experiments indicate that phosphorylation of VDAC-induced voltage-dependent closure of the channel and in vitro phosphorylation of VDAC by either glycogen synthase kinase-3β (GSK3β) or cAMP-dependent protein kinase A (PKA) increased tubulin binding and, thereby, channel occlusion [66]. Both the voltage-closed state and the tubulin-bound occluded state were reported to induce Ca^2+^ flux, and the bilayer experiments are, thus, not in line with the physiological observations of a limited Ca^2+^ uptake after PKA phosphorylation [65]. This might indicate that results from lipid bilayers are too simplistic, and an additional degree of regulation exists in vivo.

A critical link between posttranslational modifications and Ca^2+^ conductance in vivo was established in a recent study where PINK1-dependent monoubiquitinylation of VDAC1 was directly shown to limit mitochondrial Ca^2+^ uptake. Abolishing VDAC1 monoubiquitinylation by introducing a K247R mutation in VDAC1 induced a Parkinson disease phenotype in fruitflies was associated with excessive apoptosis and could be relieved by MCU knock-out [7]. Although mechanistical data on how monoubiquitinylation decreases Ca^2+^ flux are missing in this report, these data directly indicate that reducing mitochondrial Ca^2+^ uptake through VDAC1 monoubiquitinylation is a critical mechanism to suppress apoptosis.

#### 2.2.4. Modulation of VDAC Gating through the Lipidic Environment

Finally, the lipidic environment was shown to influence ion channels properties, and corresponding observations were also made for VDAC. When inserted into lipid bilayers, the addition of nonlamellar lipids such as phosphatidylethanolamine (PE) or cardiolipin induced asymmetry in voltage gating, evident by increased channel closure at negative potentials [29]. A similar effect was recently also reported for the neuroprotective cholesterol-like synthetic compound olesoxime, which also induced channel gating at more negative potentials [67]. This effect might again be relevant when VDAC is exposed to a negative potential in the OMM to facilitate or prevent gating of the channel. Additionally, the lipidic environment might also modulate the channels’ interaction with its molecular plugs. The neuroprotective cholesterol-like lipid olesoxime prevented the interaction of VDAC with α-synuclein and, thereby, affected not only VDAC voltage gating but also binding of α-synuclein to the channel [67]. As a third way to regulate VDAC through the lipidic environment, molecular dynamics simulations suggested that interactions of lipids with acidic residues of VDAC might modulate the anion selectivity of the channel [30].

Although lipid bilayer experiments and molecular dynamics simulations might not directly recapitulate channel function in vivo due to the simplicity of the system and the lack of additional regulatory factors present in its native environment, a regulation of the channel by membrane lipids might fine-tune channel activity in response to cellular stimuli also there. Indeed, a change in the membrane composition was reported, for example for the induction of apoptosis [68].

### 2.3. Regulation of VDAC Ca^2+^ Flux by Protein Partners

Many of the aforementioned mechanisms might be regulated by partner proteins. VDAC was shown to interact with many cellular proteins ranging from small signaling molecules to large enzymes and other ion channels, and these interactions are often associated with Ca^2+^ homeostasis or regulated by Ca^2+^.

In particular, members of the Bcl-2 family were described to influence mitochondrial Ca^2+^ uptake by binding to VDAC, presumably at a structure formed by the N-terminal helix and the loop connecting beta sheets 14 and 15 [36,69]. Bcl-xL was demonstrated to selectively interact with VDAC1 and VDAC3 but not VDAC2. Huang et al. report enhanced mitochondrial Ca^2+^ uptake, when Bcl-xL is associated with the channel [36] in permeabilized cells. Similarly, also the Bcl-2 member Mcl-1 interacts with VDAC1 and VDAC3 but only weakly with VDAC2 to promote the uptake of Ca^2+^ into mitochondria [70]. However, other reports in intact cells found that Bcl-xL limits Ca^2+^ uptake into mitochondria [71,72], differences that were explained by experimental disparities between whole cell experiments in which mitochondrial Ca^2+^ uptake is triggered by local IP3Rs in Ca^2+^ microdomains within contact sites of mitochondria and the SR and permeabilized cells in which mitochondrial Ca^2+^ uptake is triggered by a global rise in Ca^2+^ (see also Section 2.4). Interestingly, in lipid bilayers Bcl-xL induced a reduction in channel conductance, while preserving voltage gating [73], indicating that the Bxl-xL induced channel closure is distinct from voltage-gated closure but might also affect its Ca^2+^ conductance.

Furthermore, several enzymes have been found to interact with VDAC to regulate its Ca^2+^ flux. Among those are reports about GAPDH, which was recently shown to interact with VDAC1 to enhance the uptake of Ca^2+^ into VDAC1-containing protoliposomes [37] and the 18 kDa translocator protein TSPO, also referred to as the peripheral benzothiazepine receptor (PBR), a mitochondrial protein that forms a complex with VDAC, which, when inserted into lipid bilayers, was sensitive to its ligand hemin [74]. The addition of hemin closed VDAC and by this limited Ca^2+^ uptake into mitochondria. Although these interactions do not go into mechanistical detail, in particular, the question of why closure of the channel would decrease Ca^2+^ conductance, which is opposing to other results, they highlight the importance of direct or indirect protein–protein interactions for the regulation of VDAC.

Apart from interactions that were shown to directly alter VDAC electrophysiology or mitochondrial Ca^2+^ fluxes VDAC was repeatedly shown to interact with Ca^2+^ handling proteins, such as the SR Ca^2+^/K^+^ channel mitsugumin-23 [75], the L-type Ca^2+^ channel [76], the RyR [77], MCU [55], and the IP3R in a complex that is highly regulated through different proteins such as DJ-1 [78], grp75, or transglutaminase 2 [79]. Although these interactions were not directly shown to modulate VDAC, they still highlight its important role in Ca^2+^ handling.

Taken together, several proteins modulate VDAC directly or through higher protein complexes. However, only a small number of these reports specifically investigated Ca^2+^ conductance. Thus, ambiguity still exists about the Ca^2+^ conductivity of the gated and occluded states in vivo. Several reports indicate reduced Ca^2+^ uptake upon VDAC closure [74], while other reports do not observe changes on Ca^2+^ flux despite closure of the channel [80]. This might in part be explained by distinct modes of VDAC closure, whereof some affect Ca^2+^ conductivity and some do not, but it needs further research to selectively discriminate between them.

### 2.4. Regulation by Subcellular Localization in Ca^2+^ Microdomains

Finally, another powerful regulatory mechanism for cellular Ca^2+^ fluxes and Ca^2+^ signaling pathways is subcellular localization. In line with the idea that VDAC regulates the Ca^2+^ flux over the OMM, a distinct subcellular localization within sites of Ca^2+^ mobilization was also observed for VDAC.

Most eminently, mitochondria intimately and dynamically interact with the internal Ca^2+^ stores of the ER/SR in regions where the two organelles are densely tethered. Those sites of ER/SR-mitochondrial coupling, called mitochondrial-associated membranes (MAMs), are a key player in Ca^2+^ shuttling. MAMs cover approximately 5–20 percent of the mitochondrial surface in mammalian cells [81]. Interestingly, VDAC is not homogeneously distributed across the OMM and certain VDAC isoforms occur more frequently in MAMs and in close vicinity to ER/SR Ca^2+^ release sites, where local cytosolic Ca^2+^ can reach particularly high levels [54,77,82,83,84]. The IP3R, the Ca^2+^ release channel in the ER membrane of non-excitable cells is tethered to VDAC1 but not VDAC2 or VDAC3 [5,85], through the anchoring protein grp75 [85,86]. More recent studies have shown that in cardiomyocytes VDAC2 is analogously coupled to RyR2 [10,77]. For the subsequent transport of Ca^2+^ into mitochondria, the SR-OMM contact points align with contact points of OMM and IMM [54], and VDAC1 physically and functionally interacts with the MCUC [55].

These couplons of Ca^2+^ release (IP3R/RyR2), Ca^2+^ shuttling (VDACs), and Ca^2+^ uptake sites (MCUC) provide an efficient route for direct Ca^2+^ shuttling from high Ca^2+^ microdomains of the SR into mitochondria. They were shown to be dynamic, as they can change in response to cytosolic or extracellular triggers to regulate mitochondrial Ca^2+^ uptake [8,86] and are intensively regulated at different levels under physiological and pathophysiological conditions [8,78,87] (see Section 3 and Section 4).

However, intracellular Ca^2+^ transfer between organelles regulated through VDAC localization is not an exclusive mechanism limited to the contact sites with the ER/SR. The lysosomal Ca^2+^ release channel TRPML1 was shown to selectively interact with VDAC1 but not VDAC2 or VDAC3 to mediate Ca^2+^ transfer from lysosomes to mitochondria. Interestingly, this VDAC1-mediated lysosome–mitochondria Ca^2+^ transfer was shown to be regulated by Ca^2+^ through residue E73 [59].

**Figure 3:**
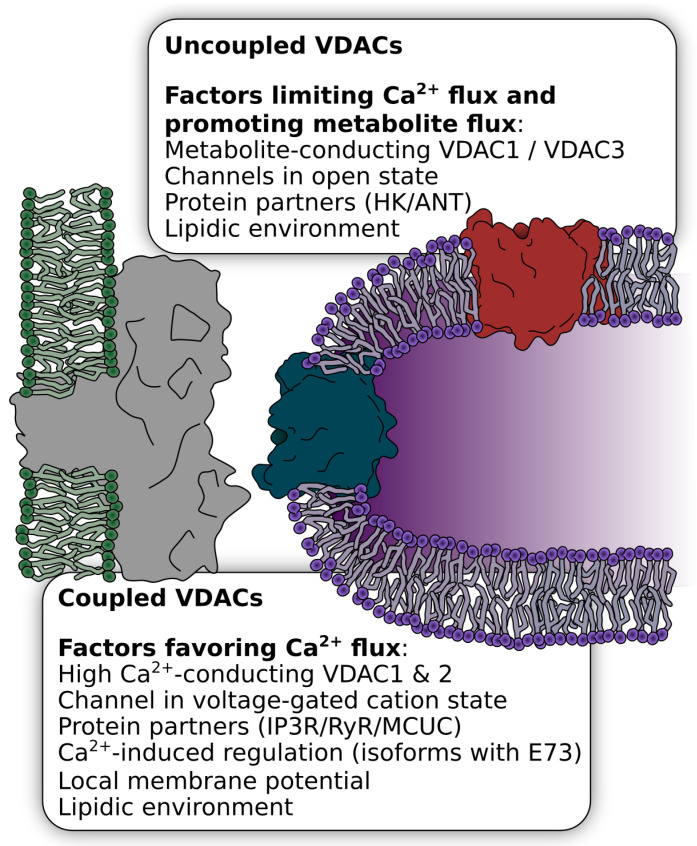
Regulation of VDAC by subcellular localization. A fraction of VDAC channels was shown to be present in couplons with Ca^2+^ release channels of the ER/SR, in particular VDAC1 with the IP3R or VDAC2 with the RyR. These couplons represent hotspots of Ca^2+^-mediated regulation of mitochondrial activity. In addition to the strategic positioning of VDAC in close vicinity to the mouth of the release channels, other factors might regulate VDAC within and outside the couplons for the specialized tasks within these environments. These include isoform selectivity, protein partners, the lipidic environment, and the voltage-gated state of the channel. Depending on the VDAC localization, these factors might determine whether VDAC serves as a Ca^2+^ or metabolite channel.

It is tempting to speculate that the subcellular localization not only positions VDAC in an ideal environment for Ca^2+^ transfer from the ER into mitochondria but also enables a higher degree of regulation. As such, it is feasible that the high Ca^2+^ concentration within this microdomain, local changes in OMM membrane potential, or a different set of partner proteins in this micro-environment allow for distinct regulation of channels inside the MAM compared to channels outside of the MAM (Figure 3).

#### 2.4.1. Regulation of the OMM Ca^2+^ Flux by Different VDAC Isoforms

An alternative but also associated form of regulation might be achieved by isoform specificity. While the three VDAC isoforms are certainly able to fulfill to some degree redundant and compensatory functions between each other, they also have different channel properties, different expression levels, and specific protein interaction partners, indicating that the isoforms serve different physiological tasks [88]. Furthermore, the same VDAC isoform might perform distinct roles in different tissues or cell types depending on its microenvironment. The specific knock-out of individual VDAC isoforms provided first insights into their function: VDAC1 KO mice are viable and studies with VDAC1^-/-^ mice confirmed that it is mainly involved in metabolite exchange and pro-apoptotic processes [89], whereof the latter is believed to be mediated by mitochondrial Ca^2+^ uptake. Indeed, in VDAC1^-/-^ HeLa cells, the transfer of local, low-amplitude Ca^2+^ signals was selectively abolished. However, mitochondrial Ca^2+^ uptake after maximum agonist response was still measurable, indicating that other VDAC isoforms, presumably outside of the IP3R couplons, are able to take up Ca^2+^ from high-amplitude signals [5].

In contrast to VDAC1, a global VDAC2 KO, was demonstrated to be lethal at around E10.5 to E11.5 of embryonic development. Furthermore, a heart-specific VDAC2 knockout showed postnatal onset of progressive fibrosis and cardiomyopathy, resulting in early mortality indicating a, presumably cardiac, role of VDAC2, which cannot be fulfilled by the two other isoforms [90]. One intriguing possibility is that the other VDAC isoforms cannot form the specific interaction of VDAC2 with RyR2 in the highly specialized heart muscle cells [77]. This would result in decreased mitochondrial Ca^2+^ uptake via RyR2 and VDAC2 and associated downstream effects such as, for example, cytosolic Ca^2+^ overload, a lack of mitochondrial energy–demand matching, or an impairment in mitochondrial function.

It is, thus, conceivable that the IP3R-VDAC1 coupling is required for the control of apoptosis through local low-amplitude Ca^2+^ signals. In cardiomyocytes, however, where local RyR-mediated Ca^2+^ signals of the SR are of a higher magnitude and more frequent, the presence of VDAC1 within these couplons would induce immediate apoptosis due its interaction with pro-apoptotic protein partners and/or other regulatory mechanisms that are specific for VDAC1. Therefore, in the heart, VDAC2 mediates a more effective flux of Ca^2+^ into mitochondria in the MAM, where it shapes cytosolic Ca^2+^ signals and rapidly accommodates energy–demand matching, while VDAC1 is located outside of the couplons and mediates metabolite flux. Indeed, VDAC2 was shown to have a different distribution of charges within the pore, which mediates a lower anion selectivity [91]. Enhanced apoptosis that might be induced by this process could be suppressed by the anti-apoptotic inhibition of BAK activation by VDAC2 [92]. The existence of IP3R-VDAC1 couplons in cardiomyocytes is under debate but would not contradict this picture.

In contrast to VDAC1 and VDAC2, the role of VDAC3 in Ca^2+^ signaling is questionable. Indeed, VDAC3 lacks residue E73 that was reported to be important for Ca^2+^-dependent regulation of the channel and is conserved in both VDAC1 and VDAC2.

#### 2.4.2. Regulation of OMM Ca^2+^ Flux by VDAC Expression Levels

A very direct way to regulate ion channel activity is the variation of the mere number of channels by adapting expression levels. Indeed, several reports have identified a variation in expression levels as a way to regulate VDAC activity. A first hint that expression levels of VDAC1 can control life and death of the cell was that downregulation of VDAC1 inhibited cell growth and overexpression lead to cell death [93]. This process seems to be physiologically relevant, since in podocytes, induction of apoptosis was shown to cause an increased expression of both VDAC1 and the IP3R, presumably to enhance mitochondrial Ca^2+^ uptake. Likewise, in diabetes, coronary endothelia cells display higher rates of apoptosis due to mitochondrial Ca^2+^ overload, which was linked to enhanced VDAC1 protein levels [94]. Vice-versa, a selective downregulation of VDAC1 but not VDAC2 was suggested to serve as a cardioprotective pathway during ischemia reperfusion [95].

Several pathways have been described to control VDAC expression. VDAC1 downregulation during ischemia reperfusion was shown to depend on liproxstatin-1 [95], and the transcription factors GATA1 and MYBL2 were reported to bind to and activate the VDAC2 promoter, to suppresses autophagy [96]. Another expression regulator could be Ca^2+^ itself. Interestingly, in a recent report, elevation of intracellular Ca^2+^ levels by Ca^2+^ mobilizing agents induced overexpression of VDAC1, while chelation of intracellular Ca^2+^ reduced VDAC1 expression [97].

## 3. Physiological Relevance of a Ca^2+^ Flux Regulation at the OMM

Mitochondria play a pivotal role in sensing, regulating, and decoding cellular Ca^2+^ signals. Mitochondrial Ca^2+^ can induce apoptotic signals and decode a “survival or death decision”, increase mitochondrial metabolism to adjust to a higher energy demand, or modulate cytosolic Ca^2+^ signals. While the main Ca^2+^ uptake route is supposed to be via MCUC in the IMM, a substantial part of the decoding could also be controlled by the permeability of the outer membrane for Ca^2+^ via VDACs and is regulated at different levels like isoform specificity, localization, and regulation of the individual channels.

One hypothesis of how VDAC mediates distinct Ca^2+^-induced signals is maybe best explained by taking highly specialized cells such as cardiomyocytes as an example. Cardiomyocytes are characterized by precisely regulated Ca^2+^ oscillations and critically depend on constant energy supply. Here, VDAC2 interacts with RyR2 in subsarcolemmal regions and at SR–mitochondria contact sites (see Section 2.4) and transfers beat-to-beat Ca^2+^ signals into mitochondria to respond to an increased workload with increased ATP synthesis. While VDAC2 is “occupied” with Ca^2+^ shuttling, VDAC1 could fulfill the function of a metabolite transporter across the outer membrane to ensure the supply of metabolites to the cytosol.

Although this is still only a hypothetical model, several experimental observations are in agreement with this split of specialized tasks between the isoforms and between coupled and uncoupled VDACs, respectively: VDAC2 was suggested to be less anion selective and, thus, more permeable to Ca^2+^. Furthermore, it was proposed that the close proximity of the IMM and OMM in the contact sites favors voltage-induced VDAC closure, which additionally favors Ca^2+^ flux, whereas VDACs that are distributed outside of these contact sites might be in an open state [25], thus favoring the flux of metabolites (Figure 3). A specific interaction of VDAC1 with ANT [98] and hexokinase (HK) might additionally favor the high-conductance state of VDAC1 and, thus, provide an effective metabolite exchange across the OMM. In this scenario, the lack of coupling of the pro-apoptotic VDAC1 with the high-efficiency Ca^2+^ release sites around RyR clusters and the anti-apoptotic features of VDAC2 prevent apoptosis induction. However, in addition to these highly efficient RyR2-VDAC2 clusters, the canonical VDAC1-IP3R clusters were also observed in cardiomyocytes [87], which might be located in areas distinct from the RyR2-VDAC2 clusters away from the SR Ca^2+^ release sites within the Z-bands and be important for mediating apoptosis.

Indeed, the VDAC1-mediated mitochondrial Ca^2+^ flux at IP3R clusters was shown to regulate apoptosis: here, VDAC1 significantly contributes to the determination of cell fate towards survival or death [99,100,101]. Mitochondrial Ca^2+^ signals can either boost mitochondrial respiration or induce apoptosis. Therefore, they need to be meticulously regulated, and it is assumed that numerous factors, in particular the interaction of VDAC1 with the IP3R, HK, and Bcl-2 family members, regulate this process. Apoptosis induction was shown to increase expression and interaction of VDAC1 with the IP3R, presumably to enhance mitochondrial Ca^2+^ uptake [5,94]. However, blocking Ca^2+^ release via IP3R or mitochondrial Ca^2+^ uptake leads to necrosis of cancer cells but not normal cells [102]. These results highlight once more the importance of Ca^2+^ signal fine tuning within nanodomains to control cellular metabolism and apoptosis but also show that more work is necessary to elucidate the exact mechanisms of VDAC-controlled mitochondrial Ca^2+^ uptake.

## 4. Regulation of VDAC-Mediated Ca^2+^ Flux under Pathophysiological Conditions

Considering the prominent role of VDAC-mediated Ca^2+^ signaling, it is only consistent that altered Ca^2+^ handling via VDAC is also associated with pathophysiology of many diseases, including cancer, cardiovascular diseases, and neurodegenerative diseases.

Cancer cell growth relies on adaptions in cell metabolism and bioenergetics, and VDACs significantly contribute to these alterations by regulating metabolite and ion flux into and out of mitochondria [103,104,105]. A central role in oncogenic behavior was ascribed to mitochondrial Ca^2+^ remodeling [106], and specifically, the VDAC1-mediated association between the ER and mitochondria was suggested to be essential for cancer cell viability. Indeed, both blocking of the IP3R Ca^2+^ release and blocking of mitochondrial Ca^2+^ uptake, respectively, lead to necrosis of cancer cells but not normal cells [102]. Strikingly, downregulation of VDAC1 via RNA interference was shown to limit cancer cell growth in vitro and tumor development in vivo without otherwise affecting the mice [107,108]. Several mechanisms were suggested to regulate VDACs Ca^2+^ conductance in cancer: hexokinase specifically interacts with VDAC1 in cancer cells to promote metabolism and to reduce apoptosis [6]. In non-small cell lung cancer cells Mcl-1, an anti-apoptotic member of the Bcl-2 family, interacts with VDAC to enhance mitochondrial Ca^2+^ uptake and ROS production resulting in an increased cancer cell migration [70].

Abnormal mitochondrial Ca^2+^ handling, resulting in energy failure, is a key factor of heart failure. Several reports describe the involvement of VDAC in this process. In ventricular cardiomyocytes isolated from failing rat hearts, the expression of the Bcl-2 family member BNIP3 was found to be significantly increased. An interaction of BNIP3 with VDAC and a consecutive shift of Ca^2+^ from the SR into mitochondria results in decreased SR Ca^2+^ content and mitochondrial damage [109]. Likewise, during remodeling after myocardial infarction, reactive aldehydes such as 4-hydroxynonenal are produced by mitochondria and by a yet not understood mechanism bind to VDAC1 and MCU to promote formation of ER–mitochondria contact sites and ultimately mitochondrial Ca^2+^ accumulation [87]. Another study showed that in diabetes, heart failure develops due to coronary microvascular rarefaction induced by apoptosis in coronary endothelia through increased VDAC1 expression [94]. Strikingly, overexpression of hexokinase 2 reduced mitochondrial overload and apoptosis [110]. Similarly, excessive VDAC-mediated Ca^2+^ transfer was also suggested in ischemia reperfusion (I/R), and a block of mitochondrial Ca^2+^ uptake was shown to be beneficial for the survival of cardiac tissue [111,112]. The close proximity between VDAC1 and SR Ca^2+^ handling proteins has been shown to be significant during I/R. Resveratrol, a polyphenolic compound in red wine, prevents VDAC1 upregulation in I/R, leading to a reduced infarct size in vivo [113]. Liproxstatin-1 was shown to be protective against I/R injury by decreasing VDAC1 expression and, thus, reducing low-amplitude apoptotic Ca^2+^ shuttling by the IP3R-Grp75-VDAC1 complex [95]. In adult mouse cardiomyocytes, the inhibition of GSK3β, which promotes an increased Ca^2+^ shuttling between IP3R and VDAC1, limits the Ca^2+^ transfer from SR/ER to mitochondria and prevents mitochondrial Ca^2+^ overload [114].

All these data indicate that mitochondrial dysfunction through Ca^2+^ overload is a key player in heart failure and I/R. However, contrarily to these results, other studies propose heart failure to be associated with a reduced ER–mitochondria coupling: in diabetes type 2, where a reduced Ca^2+^ shuttling in MAM regions was repeatedly demonstrated for many tissues [115,116,117], a reduction in IP3R-VDAC1 interaction and, thus, a decrease in IP3 stimulated Ca^2+^ transfer to mitochondria was observed for cardiomyocytes. The impaired SR–mitochondrial Ca^2+^ coupling resulted in an impairment of mitochondrial energy supply, reduced cell contraction, and thus, cardiomyopathy [118].

These data are in line with the idea that an enhanced mitochondrial Ca^2+^ uptake can be protective in pressure overload induced heart failure: TSPO closely interacts with VDAC1, acts as a negative regulator, and decreases mitochondrial Ca^2+^ uptake by inhibiting VDAC1 expression and mediating PKA phosphorylation [74,119]. Mice that underwent transverse aortic banding surgery showed significantly higher expression levels of TSPO, and cardiac TSPO-ko substantially limited the progression of pressure-induced HF and maintained cardiac function in vivo [120]. Taken together, myocardial remodeling during heart failure was repeatedly shown to affect SR–mitochondria coupling and in particular VDAC-mediated Ca^2+^ transfer; however, further research is needed to clarify opposing results of a diminished or increased ER/SR–mitochondria coupling.

Considering the important role of VDAC in physiological and especially pathophysiological modulation of mitochondrial Ca^2+^ uptake in the heart, it is, however, tempting to investigate the potential of drugs that interfere with this process. Indeed, our lab has recently described a very prominent role of the small molecular VDAC2 modulator efsevin. In lipid bilayer experiments, efsevin induced gating of VDAC2 and promoted the closed state of the channel [10]. In cardiomyocytes, this specifically increased Ca^2+^ transfer from the SR into mitochondria and, thereby, spatially and temporally restricted diastolic Ca^2+^ sparks and prevented the formation of arrhythmogenic Ca^2+^ waves [9]. Most importantly, efsevin significantly reduced ventricular tachycardia in mice.

Additionally, for neurodegenerative diseases disruption of functional ER–mitochondrial Ca^2+^ homeostasis was shown to be associated with diseases such as Parkinson’s disease (PD), Alzheimer’s disease (AD), and amyotrophic lateral sclerosis (ALS) [121]. One protein that has been associated with the early-onset of PD is DJ-1, which was recently shown to interact with the IP3R-Grp75-VDAC1 complex [78,122]. DJ-1 reduction functionally correlates with decreased mitochondrial Ca^2+^ uptake and cells expressing PD-associated DJ-1 mutants showed impaired mitochondrial function [78,123]. A recent study has further demonstrated that functional ER–mitochondria interactions are of great importance for axon regeneration after injury. The results suggest that increased Grp75 expression enhances ER–mitochondria tethering, which leads to an increase in mitochondrial Ca^2+^ uptake that seems to be beneficial for axon regeneration [124].

## 5. Conclusions

Taken together, the compiled data indicate that VDAC-mediated mitochondrial Ca^2+^ uptake, in particular, though its close interaction with the ER/SR calcium release sites, is an important modulator of physiology and pathophysiology of the cell. However, discrepancies exist between results obtained from lipid bilayer experiments and experiments performed in cells, in particular concerning the flux of Ca^2+^ upon channel closure, indicating that in the native environment other or additional levels of VDAC regulation exist and lipid bilayer experiments should not be overinterpreted. VDAC-mediated mitochondrial Ca^2+^ uptake represents a critical component to decipher cellular Ca^2+^ signals and to mediate either enhanced energy production or induction of apoptosis but also to fine-tune intracellular Ca^2+^ signals. Additionally, at least in cardiomyocytes, it further shapes cytosolic Ca^2+^ signals and acts as a buffer to blunt cytosolic Ca^2+^ spikes. VDAC-mediated Ca^2+^ uptake is regulated at different levels, ranging from expression to protein partners as well as intrinsic properties of VDAC, which can adapt multiple states of conductance. However, the specific molecular mechanisms leading to these different conductance states have not been fully resolved and care should be taken when using the term “channel closure”, since voltage-induced closure, as well as occlusion, prevents the flux of metabolites but increases Ca^2+^ flux. Future studies are needed to resolve open questions such as the role of voltage gating in vivo the relevance of VDAC gating cellular physiology and the selective contribution of the regulation of mitochondrial Ca^2+^ uptake at the OMM and IMM, respectively.
